# Depression in Individuals Coinfected with HIV and HCV Is Associated with Systematic Differences in the Gut Microbiome and Metabolome

**DOI:** 10.1128/mSystems.00465-20

**Published:** 2020-09-29

**Authors:** Bryn C. Taylor, Kelly C. Weldon, Ronald J. Ellis, Donald Franklin, Tobin Groth, Emily C. Gentry, Anupriya Tripathi, Daniel McDonald, Gregory Humphrey, MacKenzie Bryant, Julia Toronczak, Tara Schwartz, Michelli F. Oliveira, Robert Heaton, Igor Grant, Sara Gianella, Scott Letendre, Austin Swafford, Pieter C. Dorrestein, Rob Knight

**Affiliations:** a Biomedical Sciences Graduate Program, University of California San Diego, La Jolla, California, USA; b Skaggs School of Pharmacy and Pharmaceutical Sciences, University of California San Diego, La Jolla, California, USA; c Center for Microbiome Innovation, University of California San Diego, La Jolla, California, USA; d Department of Neuroscience, HIV Neurobehavioral Research Center, University of California San Diego, La Jolla, California, USA; e Department of Psychiatry, HIV Neurobehavioral Research Center, University of California San Diego, La Jolla, California, USA; f Department of Psychiatry, School of Medicine, University of California San Diego, La Jolla, California, USA; g Division of Biological Sciences, University of California San Diego, La Jolla, California, USA; h Department of Pediatrics, School of Medicine, University of California San Diego, La Jolla, California, USA; i Department of Medicine, University of California San Diego, La Jolla, California, USA; j Division of Infectious Diseases and Global Public Health, University of California San Diego, La Jolla, California, USA; k Department of Psychiatry, University of California San Diego, La Jolla, California, USA; l Department of Computer Science and Engineering, University of California San Diego, La Jolla, California, USA; m Department of Bioengineering, University of California San Diego, La Jolla, California, USA; Princeton University

**Keywords:** HIV, hepatitis C, microbiome, depression, gut microbiome, hepatitis C virus, human immunodeficiency virus

## Abstract

Depression is influenced by the structure, diversity, and composition of the gut microbiome. Although depression has been described previously in human immunodeficiency virus (HIV) and hepatitis C virus (HCV) monoinfections, and to a lesser extent in HIV-HCV coinfection, research on the interplay between depression and the gut microbiome in these disease states is limited. Here, we characterized the gut microbiome using 16S rRNA amplicon sequencing of fecal samples from 373 participants who underwent a comprehensive neuropsychiatric assessment and the gut metabolome on a subset of these participants using untargeted metabolomics with liquid chromatography-mass spectrometry. We observed that the gut microbiome and metabolome were distinct between HIV-positive and -negative individuals. HCV infection had a large association with the microbiome that was not confounded by drug use. Therefore, we classified the participants by HIV and HCV infection status (HIV-monoinfected, HIV-HCV coinfected, or uninfected). The three groups significantly differed in their gut microbiome (unweighted UniFrac distances) and metabolome (Bray-Curtis distances). Coinfected individuals also had lower alpha diversity. Within each of the three groups, we evaluated lifetime major depressive disorder (MDD) and current Beck Depression Inventory-II. We found that the gut microbiome differed between depression states only in coinfected individuals. Coinfected individuals with a lifetime history of MDD were enriched in primary and secondary bile acids, as well as taxa previously identified in people with MDD. Collectively, we observe persistent signatures associated with depression only in coinfected individuals, suggesting that HCV itself, or interactions between HCV and HIV, may drive HIV-related neuropsychiatric differences.

**IMPORTANCE** The human gut microbiome influences depression. Differences between the microbiomes of HIV-infected and uninfected individuals have been described, but it is not known whether these are due to HIV itself, or to common HIV comorbidities such as HCV coinfection. Limited research has explored the influence of the microbiome on depression within these groups. Here, we characterized the microbial community and metabolome in the stools from 373 people, noting the presence of current or lifetime depression as well as their HIV and HCV infection status. Our findings provide additional evidence that individuals with HIV have different microbiomes which are further altered by HCV coinfection. In individuals coinfected with both HIV and HCV, we identified microbes and molecules that were associated with depression. These results suggest that the interplay of HIV and HCV and the gut microbiome may contribute to the HIV-associated neuropsychiatric problems.

## Abstract (precis)

The human gut microbiome influences depression. Differences between the microbiomes of HIV-infected and uninfected individuals have been described, but it is not known whether these are due to HIV itself, or to common HIV comorbidities such as HCV coinfection. Limited research has explored the influence of the microbiome on depression within these groups. Here, we characterized the microbial community and metabolome in the stools from 373 people, noting the presence of current or lifetime depression as well as their HIV and HCV infection status. Our findings provide additional evidence that individuals with HIV have different microbiomes which are further altered by HCV coinfection. In individuals coinfected with both HIV and HCV, we identified microbes and molecules that were associated with depression. These results suggest that the interplay of HIV and HCV and the gut microbiome may contribute to the HIV-associated neuropsychiatric problems.

## INTRODUCTION

Disturbances in gut microbial communities may contribute to depression and neuropsychiatric disorders in human immunodeficiency virus (HIV) infection (1–3). Depletion of CD4^+^ T cells in gut lymphoid tissue occurs very early in HIV infection and is associated with dysbiosis and gut barrier dysfunction (“leaky gut”) (4, 5), which are not normalized by virologic suppression on antiretroviral therapy (ART) (6). Leaky gut in HIV infection is associated with increased apoptosis, chronic inflammatory signals, and reduced proliferation and repair of epithelial cells (4, 5, 7, 8) which may further introduce microbial metabolites known to impact brain activity (1, 9–11). Gut dysbiosis patterns in HIV monoinfection may include greater proportions of Gram-negative bacteria, order *Enterobacteriales* (12), enrichment of *Proteobacteria* (13), depletion of *Bacteroidia* (14) and increased abundances of *Prevotellaceae* and *Erysipelotrichaceae* (15). Some of these alterations involve proinflammatory species (e.g., *Prevotella*) (16–18). Together dysbiosis and leaky gut render HIV-infected individuals more vulnerable to microbial antigen-driven effects on the central nervous system (CNS) via proinflammatory bacterial antigens such as lipopolysaccharide (LPS) and flagellin (19, 20).

Dysbiosis-driven inflammation also may lead to depression, as suggested by existing literature (21, 22). The gut microbiome may affect blood-brain barrier (BBB) integrity as well (23), and this may potentiate depression (24–26). For example, germfree mice have reduced expression of tight junction proteins on brain microvascular endothelial cells. BBB integrity was restored after gut colonization or by administration of butyrate (27). BBB compromise may amplify entry of HIV and associated neurotoxins into the CNS (28). These findings are of clinical importance, since interventions exist to restore normal gut microbes and barrier integrity (such as Bacteroides fragilis or Bacteroides thetaiotaomicron polysaccharide A [PSA] [29], butyrate [30], and tryptophan metabolites [31]) with the potential to improve CNS function.

While no systematic research has been reported on the impact of HIV-HCV coinfection on the gut microbiome, a number of reports examining very different cohorts of patients with HCV monoinfection have evaluated alterations in the gut microbiome. A study of HCV patients with advanced liver disease showed that they exhibited increased abundance of *Bacteroidetes* and *Firmicutes* compared to healthy subjects (32). The HCV patients had increased *Prevotella*, Acinetobacter, *Veillonella*, *Phascolarctobacterium*, and *Faecalibacterium* and reduced *Ruminococcus*, *Clostridium*, and *Bifidobacterium* genus. Interpreting these findings is difficult, as these patients were likely treated with luminal antibiotics as prophylaxis against hepatic encephalopathy (32). In another study of persons with HCV, bacterial diversity was lower compared with healthy individuals, with reduced *Clostridiales* and increased *Streptococcus* and *Lactobacillus*. Dysbiosis appeared very early, before cirrhotic changes (33). In another report, gut microbiome alpha diversity was reduced in cirrhotic patients, but dysbiosis was significantly improved along with a reduction in serum cytokines and chemokines by curing HCV infection after treatment with direct-acting agents (34). However, another study showed that cirrhotic outpatients with HCV had similar microbiome and proinflammatory changes before and 1 year after HCV cure (35). Thus, there is no consensus concerning changes in the gut microbiome associated with HCV, likely due to marked differences in the cohorts studied.

Abundant human and animal evidence link the gut microbiome to neuroinflammation and depressed mood. In rats treated with microbiota from rats vulnerable to social stress, there was higher microglial density and interleukin-1β (IL-1β) expression in the ventral hippocampus and higher depression-like behaviors relative to rats receiving microbiota from rats resistant to social stress, suggesting that the gut microbiome contributes to the depression-like behavior and inflammatory processes in the brain (36). In HIV-positive individuals, an abnormal microbiome in combination with leaky gut leads to high circulating levels of microbial antigens that provoke inflammation. This inflammation induces expression of indoleamine dioxygenase, which promotes depressed mood by shunting tryptophan away from serotonin synthesis (37).

Similarly, in humans without these infections, the gut microbiome can influence neuroinflammation and neuropsychiatric disorders through the gut-brain axis (38). For example, patients with major depressive disorder (MDD) showed increased *Bacteroidetes*, *Proteobacteria*, and *Actinobacteria* and reduced *Firmicutes* (39). Interventions that affect the gut microbiome can be beneficial for neuropsychiatric dysfunction. For example, probiotics and prebiotics attenuated the physiological stress response: colonizing germfree male mice with Bifidobacterium infantis normalized their previously overreactive hypothalamic-pituitary-adrenal axis in response to restraint stress (40). Also, treatment with prebiotic fructo- and galacto-oligosaccharides (FOS/GOS) lowered proinflammatory cytokine levels in mice exposed to chronic psychosocial stress (41).

To address gaps in knowledge about the impact of coinfection with HIV and HCV on the gut microbiome, we performed 16S rRNA sequencing and metabolomics analyses on fecal samples from coinfected individuals and compared them to HIV-monoinfected and HIV uninfected subjects. Despite the evident interplay between HIV infection and associated neurocognitive disorders, and between each of these and gut microbiome dysbiosis, prior work suggests that HIV infection and neurocognitive disorders are not associated with gut microbiome dysbiosis (42). Here, we observe associations between gut microbiome dysbiosis and depression, a form of neurobehavioral disorder, only in HIV-HCV coinfected individuals. These results suggest that HIV, HCV, and the gut microbiome may work together to cause neuropsychiatric problems associated with HIV.

## RESULTS AND DISCUSSION

### The gut microbiome and metabolome differ with HIV and HCV infection.

We first evaluated the gut microbiome and metabolome in the context of HIV infection status. As in previous studies (43–51), we found that beta diversity (i.e., between subject) differed between HIV-positive (*n* = 267) and -negative individuals (*n* = 106) (unweighted UniFrac distances [52–54]: permutational multivariate analysis of variance [PERMANOVA] pseudo-F-statistic [pseudo-F] = 4.24, Benjamini-Hochberg-corrected [BH] *P* = 0.001). However, we found that alpha diversity (i.e., within subject) did not differ between HIV-positive and -negative individuals. There was also a significant difference in the gut metabolome between HIV-positive and -negative individuals (Bray-Curtis, pseudo-F = 5.82, BH *P* = 0.001).

**FIG 1:**
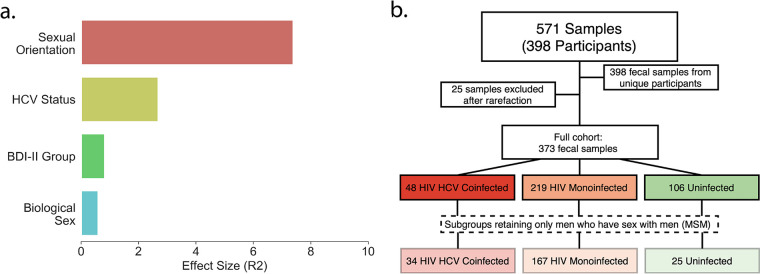
Cohort characteristics. (a) Unweighted UniFrac relative effect sizes assessed using RDA in the full data set. (b) Sample selection pipeline. Coinfected groups (red), HIV-monoinfected groups (orange), and uninfected groups (green) are indicated. Lighter colors represent MSM subgroups.

To characterize the impact of covariates on the microbiome, we performed regularized discriminant analysis (RDA) (55) to calculate the relative effect size of several covariates: sexual orientation, biological sex, HCV status, HIV status, Beck Depression Inventory-II (BDI-II) group, lifetime alcohol use disorders, lifetime MDD, and lifetime drug use disorders (including lifetime history of cocaine, methamphetamine, heroin, and sedative use disorders) in the unweighted UniFrac beta diversity principal coordinate analysis (PCoA). The lifetime drug use disorder categories were colinear with each other in the PCoA, but no drug use disorder category was colinear with HCV infection status. This suggests that a history of drug use disorders does not confound HCV status. After merging colinear drug use disorder covariates, we found that sexual orientation, HCV infection status, BDI group (mild ≤ 13, 13 to 19, mild; 20 to 28, moderate; or > 28 severely depressed current mood), and biological sex resulted in a significant RDA model (Fig. 1a).

Due to the large relative effect size of HCV status, we classified the participants by the presence or absence of both HIV and HCV infection (HIV monoinfected, HIV-HCV coinfected, or uninfected (Table 1, MSM subset). In the coinfected group, only 26% had any one or more laboratory values, suggesting active HCV infection (serum alanine transaminase [ALT] > 55, aspartate aminotransferase [AST] > 40, total bilirubin > 1.2, direct bilirubin > 0.3, or APRI [AST-to-platelet ratio index] > 1.0), compared to 18.2% of HIV-monoinfected and 10% of uninfected individuals. Only one HCV seropositive individual had been treated with an anti-HCV direct-acting antiviral, consistent with a treatment era in which all HCV seropositive individuals with active disease are offered curative anti-HCV treatment. Thus, the proportion of coinfected participants with active HCV disease was likely to be negligible.

**TABLE 1:** HIV and HCV group characteristics for both the full infection cohorts and MSM subset^*a*^

Characteristic^*b*^	Value for the following subgroup of the full cohort:			Sign. diff. in the full cohort^*c*^	Value for the following subgroup of the MSM subset:			Sign. diff. in the MSM subset^*c*^
	Uninfected (a) (*n* = 106)	HIV-mono- infected (b) (*n* = 219)	Coinfected (c) (*n* = 48)		Uninfected (a) (*n* = 25)	HIV-mono- infected (b) (*n* = 167)	Coinfected (c) (*n* = 34)	
Age^*d*^	51.2 (16.3)	51.7 (12.0)	53.8 (9.1)		52.8 (17.1)	51.2 (12.7)	52.9 (9.0)	
Education^*d*^	14.4 (2.5)	14.2 (2.5)	13.5 (2.6)		15.0 (2.4)	14.5 (2.4)	13.5 (2.5)	a > c
% female	40	12	15	a > b,c				
% Caucasian	56	58	40	b > c	64	63	46	
Est. verbal IQ^*d*^	104.8 (15.5)	102.0 (12.5)	98.7 (13.8)	a > c	110.7 (17.4)	103.0 (11.4)	101.2 (14.0)	a > b,c
Sexual orientation (%)								
Bisexual	6	8	19	a,b < c	20	10	24	b < a,c
Heterosexual	72	20	26	a > b,c				
Homosexual	22	71	55	a < c < b	80	90	76	
Other/not asked	0	1	0					
% AIDS		59	73			57	71	
Est. duration HIV+ (yrs)^*d*^		17.5 (9.9)	21.5 (7.6)	b < c		17.8 (10.4)	21.9 (8.2)	b < c
Nadir CD4^*e*^		178 [24−214]	149 [12−284]			183 [40−343]	175 [14−300]	
Current CD4^*e*^		630 [456−840]	521 [420−794]			627 [440−820]	508 [357−739]	
% undetectable HIV RNA (plasma; on ART)		93	89			92	93	
% on ART		96	85	b > c		95	86	b > c
% cognitively impaired	44	49	56		42	48	55	
Beck Depression Inventory-II^*d*^	5.6 (7.3)	11.2 (10.8)	10.9 (10.7)	a < b,c	6.7 (7.0)	11.3 (11.0)	10.3 (10.4)	
% employed	43	32	19	a > c	38	31	23	
% IADL dependent	10	37	48	a < b,c	12	34	48	a < b,c
% lifetime substance use disorder	55	74	85	a < b,c	76	76	86	
% lifetime major depressive disorder (MDD)	33	52	71	a < b < c	32	52	63	a < b < c
% of lifetime MDD on an antidepressant	15	52	44	a < b,c	0	56	41	a < b,c
% of lifetime MDD on an SSRI	12	22	12		0	26	9	
% of lifetime MDD on an SNRI	3	16	15		0	15	14	

a *t* tests were used for all normally distributed continuous variables (age, education, estimated verbal IQ, estimated duration HIV, Beck Depression Inventory-II). Wilcoxon tests were used for nadir and current CD4. Chi-square tests were used for all nominal variables (percent Caucasian, percent AIDS, percent undetectable HIV RNA, percent cognitively impaired, percent employed, percent IADL dependent, percent lifetime substance use disorder, percent lifetime major depressive disorder, percent bisexual and/or homosexual, percent lifetime MDD on an antidepressant, percent lifetime MDD on an SSRI, percent lifetime MDD on an SNRI).

b Est., estimated; HIV+, HIV positive; IADL, instrumental activities of daily living.

c The Sign. diff columns indicate whether the groups (groups a, b, and c) show a significant difference (alpha = 0.05) and in which direction.

d Mean (standard deviation) shown for these characteristics.

e Median [interquartile range {IQR}] shown for these characteristics.

### Demographic and lifestyle comparisons between coinfected, HIV-monoinfected, and uninfected individuals.

To explore the relationship between the gut microbiome and depression in people with HIV monoinfection, HIV and HCV coinfection, or neither, we analyzed 16S rRNA gene amplicon sequencing data from a total of 571 fecal samples (Fig. 1b), 398 of which were from unique individuals. After filtering (see Materials and Methods), 373 samples from unique subjects (described in Table 1, full cohort) were retained for analysis. Participants were grouped according to their HIV and HCV infection state: “coinfected” individuals (*n* = 48) with both HIV and HCV, “HIV monoinfected” (*n* = 219) with HIV but not HCV, and “uninfected” individuals (*n* = 106) with neither virus. A subset of these participants (coinfected, *n* = 27; HIV monoinfected, *n* = 82; uninfected, *n* = 32) were additionally assessed using untargeted metabolomics by liquid chromatography-mass spectrometry.

Sample characteristics of each infection group are included in Table 1. Biological sex, anal receptive intercourse, and age have been associated with differences in microbial communities (56–66). The uninfected group had more women and significantly fewer bisexual and homosexual men (χ^2^ = 76.9, *P* < 0.0001) than the other infection groups, but the three groups were similar in terms of age. The uninfected group had a higher estimated verbal IQ than the coinfected group. The uninfected group also had lower current depressive symptoms and fewer problems with activities of daily living than the HIV-monoinfected and coinfected groups and a higher rate of employment than the coinfected group. Lifetime substance use disorders were lower in the uninfected group, while lifetime major depression showed a stair step pattern with the uninfected group at 33%, the HIV-monoinfected group at 52%, and the coinfected group at 71% (all *P* values < 0.05). In terms of HIV disease, the HIV-monoinfected and coinfected groups did not differ by AIDS status, current or nadir CD4, or plasma viral load detectability. While the coinfected group had been HIV positive an average of 4 years longer than the HIV-monoinfected group, they were less likely to be on ART at their study visit (85% versus 96%, respectively; *P* < 0.01). The coinfected group was also more likely to be composed of minorities (specifically, African-Americans), but in all other respects (including history of substance use disorders), the individuals in the coinfected group were comparable to the individuals in the HIV-monoinfected group.

A subset of participants with a history of MDD were taking antidepressants at the time of evaluation, which may have the capacity to alter gut microbiome and metabolome composition. Of the participants with a lifetime history of MDD, 26% of the coinfected, 34% of the HIV monoinfected, and 15% of the uninfected were taking antidepressants, which included selective serotonin reuptake inhibitors (SSRIs) or serotonin-norepinephrine reuptake inhibitors (SNRIs) (Table 1). In addition, because diet can directly affect the gut microbiome, a subset of the participants in the full cohort (coinfected, *n* = 7; HIV monoinfected, *n* = 39; uninfected, *n* = 34) completed a dietary intake survey (see Table S1 in the supplemental material). Of the 22 food categories surveyed, there was only a significant difference in consumption of “home-cooked meals” between the three infection groups, such that the coinfected group was less likely to report regular to daily consumption (3 to 7 days/week) of home-cooked meals than both the uninfected and HIV-monoinfected groups (χ^2^ = 20.15, *P* < 0.05).

10.1128/mSystems.00465-20.1 TABLE S1 Dietary intake comparison using chi-square tests between the full infection groups. The final column indicates if there is a significant difference between groups (*P* < 0.05) and in which direction. Download Table S1, CSV file, 0.00 MB. Copyright © 2020 Taylor et al. 2020 Taylor et al. This content is distributed under the terms of the Creative Commons Attribution 4.0 International license.

Men who have sex with men (MSM) are known to have *Prevotella*-rich gut microbiomes, which is also a hallmark in HIV infection (59–66). To account for this potentially confounding factor, we performed concerted microbiome analyses on (i) the full groups (coinfected, HIV monoinfected, uninfected) and (ii) the subgroups composed only of MSM (coinfected, *n* = 34; HIV monoinfected, *n* = 167; uninfected, *n* = 25; Fig. 1).

When limited to MSMs, the uninfected group had somewhat higher education levels than the coinfected group, and higher premorbid IQ estimates than both infected groups; otherwise, demographic characteristics did not differ between the three subgroups (Table 1, MSM subset). The three groups differed in sexual behavior (χ^2^ = 7.7, *P* = 0.02): in the coinfected subgroup, 24% reported sex with men and women and 76% reported sex with only men; for the HIV-monoinfected subgroup, 10% reported sex with men and women and 90% reported sex with only men. In the uninfected subgroup, 20% reported sex with men and women and 80% reported sex with only men. As in the full group, the coinfected individuals in the MSM subgroup had longer estimated duration of HIV infection and a smaller percentage were on antiretroviral therapy (ART) in comparison to the HIV-monoinfected MSM subgroup. Additional cohort descriptors are included in Table 1. Of the MSM data set, 20 coinfected, 67 HIV-monoinfected, and 8 uninfected individuals were assessed using untargeted mass spectrometry.

### The gut microbiome and metabolome are significantly different between coinfected, HIV-monoinfected, and uninfected individuals.

To understand how the gut microbiome and metabolome of the three infection groups differed from each other, we compared alpha and beta diversity between coinfected, HIV-monoinfected, and uninfected groups. After examining results with the full cohort, we then performed the same analyses on the MSM subgroups.

**FIG 2:**
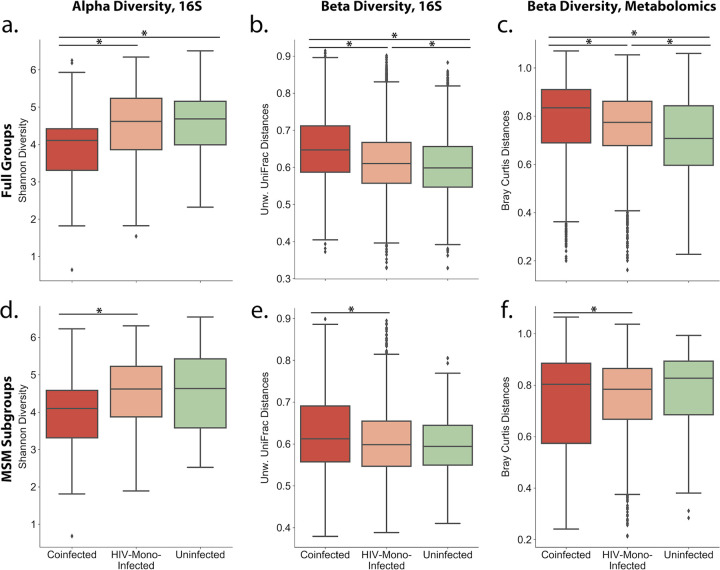
Comparison between coinfected (red), HIV-monoinfected (orange), and uninfected (green) groups. (a to c) Full groups. (d to f) MSM subgroups. (a and d) Between-group alpha (Shannon index) diversity compared to the uninfected group, compared using Kruskal-Wallis test; (b and e) between-group unweighted UniFrac distances of microbiome profiles, compared to the uninfected group, compared using pairwise PERMANOVA; (C and f) between-group Bray-Curtis distances of metabolomic profiles compared to the uninfected group, compared using pairwise PERMANOVA. The false discovery rate (FDR) was controlled using the Benjamini-Hochberg procedure.

First, we compared coinfected to uninfected groups. In the full cohort, we observed a statistically significant difference in the overall gut microbial communities in unweighted UniFrac beta diversity distances between coinfected and uninfected individuals (Fig. 2a, PERMANOVA pseudo-F = 3.05, BH *P* = 0.001). Coinfected individuals also had lower alpha diversity than uninfected individuals (Fig. 2b, Shannon index [67], Kruskal-Wallis H [KW-H] = 14.0, BH *P* = 0.0006). Coinfected and uninfected individuals were also significantly different in their overall gut metabolome (Fig. 2c, beta diversity Bray-Curtis PERMANOVA pseudo-F = 7.57, BH *P* = 0.002). However, between coinfected and uninfected MSM subgroups, there were no differences in the overall composition of the gut microbiome and metabolome (unweighted UniFrac beta diversity PERMANOVA pseudo-F = 1.28, BH *P* = 0.20; Shannon index, KW-H = 2.85, BH *P* = 0.14; metabolomics beta diversity Bray-Curtis PERMANOVA pseudo-F = 0.98, BH *P* = 0.47).

Next, we compared HIV-monoinfected to uninfected groups. HIV-monoinfected individuals were also significantly different from uninfected individuals in unweighted UniFrac beta diversity distances (Fig. 2a, PERMANOVA pseudo-F = 4.4, BH *P* = 0.001), and in their overall gut metabolome (Fig. 2c, beta diversity Bray-Curtis PERMANOVA pseudo-F = 4.53, BH *P* = 0.002). Unlike coinfected individuals, however, there was no difference in alpha diversity between the HIV-monoinfected and uninfected groups (Shannon index, KW-H = 0.37, BH *P* = 0.55). Between the MSM subgroups of HIV-monoinfected and uninfected groups, there were no differences in the overall composition of the gut microbiome and metabolome (unweighted UniFrac beta diversity PERMANOVA pseudo-F = 1.18, BH *P* = 0.21; Shannon index, KW-H = 0.0006, BH *P* = 0.98; metabolomics beta diversity Bray Curtis PERMANOVA pseudo-F = 0.92, BH *P* = 0.47).

Finally, we compared coinfected to HIV-monoinfected groups. In the full cohorts, we observed a statistically significant difference in unweighted UniFrac beta diversity distances between coinfected and HIV-monoinfected individuals (Fig. 2a, PERMANOVA pseudo-F = 2.56, BH *P* = 0.001). Coinfected individuals also had lower alpha diversity than HIV-monoinfected individuals (Fig. 2b, Shannon index, KW-H = 12.5, BH *P* = 0.0006). Furthermore, coinfected and HIV-monoinfected individuals were significantly different in their overall gut metabolome (Fig. 2c, beta diversity Bray-Curtis PERMANOVA pseudo-F = 3.416891, BH *P* = 0.004). In the MSM subgroups, the unweighted UniFrac beta diversity distances between the coinfected and HIV-monoinfected subgroups remained statistically significantly different (Fig. 2d, PERMANOVA pseudo-F = 1.73, BH *P* = 0.05). Again, the coinfected individuals had a lower alpha diversity than HIV-monoinfected individuals (Fig. 2e, Shannon index, KW-H = 6.38, BH *P* = 0.04). The differences in the overall gut metabolomes of the coinfected and HIV-monoinfected individuals also remained significant in the MSM cohort (Fig. 2f, beta diversity, Bray-Curtis PERMANOVA pseudo-F = 3.15, BH *p* = 0.03).

### Alpha diversity does not correlate with immune biomarkers of disease progression in each cohort.

Progression of untreated HIV infection is associated with worsening immune suppression, which is characterized by lower CD4^+^ T-cell counts and higher CD8^+^ T-cell counts (68), resulting in a low CD4/CD8 ratio. We did not observe any correlation between percent CD4^+^, nadir CD4^+^, or absolute CD4^+^ T cells, and alpha diversity (Shannon) in any of the infection groups (Table S2). Likewise, there was no correlation between CD4/CD8 ratio and alpha diversity (Shannon index) (Table S2).

10.1128/mSystems.00465-20.2 TABLE S2 Alpha (Shannon index) diversity correlations with continuous BDI-II and continuous biomarkers in the infection groups and MSM subgroups. Download Table S2, CSV file, 0.00 MB. Copyright © 2020 Taylor et al. 2020 Taylor et al. This content is distributed under the terms of the Creative Commons Attribution 4.0 International license.

Elevated levels of the proinflammatory cytokine interleukin-6 (IL-6), even in the context of viral suppression on ART, are associated with adverse outcomes such as myocardial infarction and death (69–72). There were no correlations between plasma IL-6 and alpha diversity (Shannon) in any of the infection groups or subgroups (Table S2).

### Associations of gut microbiome and metabolome composition with current and lifetime depression within the coinfected, HIV-monoinfected, and uninfected cohorts.

We next evaluated each of the three infection groups separately to assess associations between the gut microbiome and depression. Participants underwent standardized assessments of lifetime major depressive disorder (MDD) using *Diagnostic and Statistical Manual of Mental Disorders*, 4th edition (DSM-IV) (73) criteria (and current depressive symptoms using the Beck Depression Inventory-II) as described in Materials and Methods. Here, we evaluated the groups according to two assessments: occurrence of lifetime MDD and current depressive symptoms of at least mild severity based on the Beck Depression Inventory (BDI-II ≥ 14).

### The gut microbiome and metabolome are altered in coinfected individuals with depression.

We first tested for association between the gut microbiome and BDI-II in any of the three groups. Individuals were considered currently depressed if they reported at least mild depressive symptoms; otherwise they were considered not depressed. In no infection cohort was there a significant difference in alpha or beta diversity between individuals stratified by current depressive symptoms (Table S3). Consistent with prior research (74, 75), there also was no significant correlation between alpha diversity and continuous BDI-II severity in any of the cohorts (Table S2).

10.1128/mSystems.00465-20.3 TABLE S3 Alpha and beta diversity between categorical BDI-II and lifetime (LT) MDD groups in all infection groups. Beta (unweighted UniFrac) and alpha (Shannon) diversity in the 16S data, and beta (Bray-Curtis) diversity in the metabolomics data. Download Table S3, CSV file, 0.00 MB. Copyright © 2020 Taylor et al. 2020 Taylor et al. This content is distributed under the terms of the Creative Commons Attribution 4.0 International license.

We were also interested in determining whether having MDD at any point (or multiple points) in an individual’s life would be associated with gut microbiome differences, separately within the three infection groups. Only in the full coinfected group did we observe a statistically significant difference between those who met lifetime diagnostic criteria for MDD versus those who did not (Table S3, unweighted UniFrac PERMANOVA, pseudo-F = 1.6, BH *P* = 0.044). We found no significant differences in the HIV-monoinfected full group or MSM subgroup in unweighted UniFrac distances or Shannon diversity between MDD states (Table S3). Prior research also suggests that neurobehavioral disorders are not independently associated with gut microbiome dysbiosis in HIV infection (42). We also found no significant differences between lifetime MDD status in the uninfected groups (Table S3).

**FIG 3:**
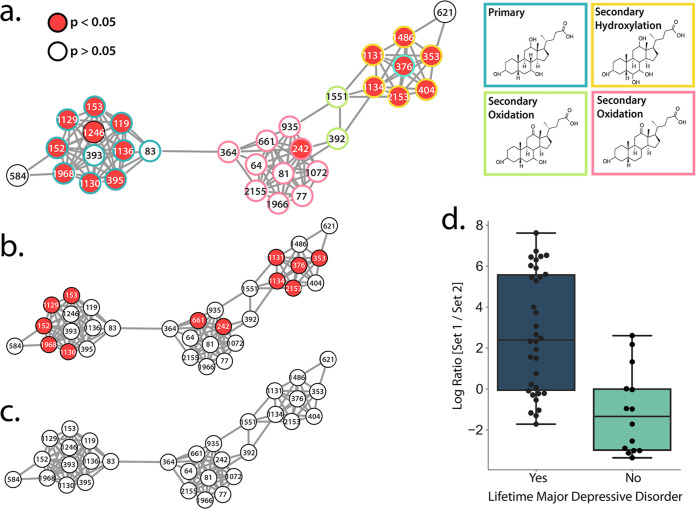
The gut microbiome and metabolome differ in coinfected individuals with a lifetime history of MDD. (a to c) Bile acid networks. Red indicates that the bile acid was significantly higher (Dunn’s test, *P* < 0.05) in individuals who had a lifetime history of MDD versus those who never had MDD. The list of GNPS annotations for this network are available in Table S7 in the supplemental material. (a) Full coinfected cohort; primary and secondary bile acid annotation of the network. (b) Coinfected MSM subgroup. (c) All other cohorts. (d) Individuals who had lifetime MDD had a significantly higher log ratio of set 1 to set 2 (*t* test, *P* = 9.1e−06, t = −5.21, df = 34.18, Cohen’s D = 1.43). The list of microbes in each set are available in Table S8.

In the metabolomics data, a random forest analysis was used to identify features of interest between lifetime MDD status within each infection group. The top 50 features of importance found in each infection group using this machine learning analysis can be found in Tables S4, S5, and S6. These features and their spectral matches (if present) can be found in the Global Natural Products Social Molecular Networking (GNPS) feature-based molecular networking job (https://gnps.ucsd.edu/ProteoSAFe/status.jsp?task=350392e8e24c41f2b84fde04f9183fc4). Multiple compounds of interest were found to be annotated as bile acids. Further analysis of all annotated bile acids revealed that in both the full coinfected group and the coinfected MSM subgroup, a cluster of primary and secondary bile acids were significantly increased (Dunn’s test, *P* < 0.05) in individuals with a lifetime history of MDD (Fig. 3a and b; annotations in Table S7). This difference was not observed in the HIV-monoinfected or uninfected group (Fig. 3c).

10.1128/mSystems.00465-20.4 TABLE S4 QIIME2 sample classifier list of metabolomics features of importance in the HIV-HCV coinfected individuals when discriminating between lifetime MDD status. All annotations are MSI level 2 or 3. The bold text indicates features contained in the bile acid networks of interest in Fig. 3a to c. Download Table S4, CSV file, 0.00 MB. Copyright © 2020 Taylor et al. 2020 Taylor et al. This content is distributed under the terms of the Creative Commons Attribution 4.0 International license.

10.1128/mSystems.00465-20.5 TABLE S5 QIIME2 sample classifier list of metabolomics features of importance in the HIV monoinfected individuals when discriminating between lifetime MDD status. All annotations are MSI level 2 or 3. The bold text indicates features contained in the bile acid networks of interest in Fig. 3a to c. Download Table S5, CSV file, 0.00 MB. Copyright © 2020 Taylor et al. 2020 Taylor et al. This content is distributed under the terms of the Creative Commons Attribution 4.0 International license.

10.1128/mSystems.00465-20.6 TABLE S6 QIIME2 sample classifier list of metabolomics features of importance in the uninfected individuals when discriminating between lifetime MDD status. All annotations are MSI level 2 or 3. The bold text indicates features contained in the bile acid networks of interest in Fig. 3a to c. Download Table S6, CSV file, 0.00 MB. Copyright © 2020 Taylor et al. 2020 Taylor et al. This content is distributed under the terms of the Creative Commons Attribution 4.0 International license.

10.1128/mSystems.00465-20.7 TABLE S7 GNPS annotations (MSI level 3) for the bile acid networks shown in all figures. The annotation indicates the closest spectral match found in the GNPS libraries for the feature. Download Table S7, CSV file, 0.00 MB. Copyright © 2020 Taylor et al. 2020 Taylor et al. This content is distributed under the terms of the Creative Commons Attribution 4.0 International license.

10.1128/mSystems.00465-20.8 TABLE S8 Sets of taxa identified using Songbird and used in log ratio calculations. Download Table S8, CSV file, 0.00 MB. Copyright © 2020 Taylor et al. 2020 Taylor et al. This content is distributed under the terms of the Creative Commons Attribution 4.0 International license.

Bile acids and the gut microbiome exist in a dynamic equilibrium (76). Primary bile acids are produced and conjugated in the liver, released in the biliary tract, and maintained through positive-feedback antagonism of farnesoid X receptor (FXR) in the gut and liver (77). Bile acids mediate anti-inflammatory immune responses by binding to receptors such as Takeda G-protein-coupled receptor 5 (78, 79). Primary bile acids are metabolized by gut microbes into secondary bile acids and passively absorbed into the portal circulation (80). Secondary bile acids affect host physiology by binding and activating host nuclear receptors to a greater extent than primary bile acids (76). Here, seven annotations of secondary bile acids were significantly increased in the full cohort of coinfected individuals with a lifetime history of MDD, and six were significantly increased in the MSM subgroup (Fig. 3a and b). This finding suggests increased metabolism of primary to secondary bile acids by gut microbes in individuals coinfected with both HIV and HCV.

Bile acid imbalances are known to be associated with pathological states such as liver disease, gastrointestinal cancers, and gallstones (76). Shifts in bile acid homeostasis are associated with HCV infection (81) and chronic liver disease. Bile acid abundance and composition are also dysregulated in MDD (82). Our observation of increased primary and secondary bile acids in coinfected individuals with a lifetime history of MDD compared to coinfected individuals without a lifetime history of MDD suggests that dysregulated bile acid metabolism by gut bacteria may be a mechanism that links HIV-HCV coinfection and MDD.

Due to our observation that overall microbiome composition, as measured by unweighted UniFrac distances, differed between coinfected individuals with or without lifetime MDD, we were also interested in determining whether specific groups of taxa may be driving the bile acid differences we observed in the gut metabolome. We used Songbird (83) to identify microbes that were associated with lifetime MDD in the full cohort of coinfected individuals. Songbird is a compositionally aware differential abundance method which provides rankings of features (suboperational taxonomic units [sOTUs]) based on their log fold change with respect to covariates of interest. In this case, the formula we used described whether the individual had lifetime MDD or not. We selected the highest 10% (“set 1” in Table S8) and lowest 10% (“set 2” in Table S8) of the ranked sOTUs associated with lifetime MDD and used Qurro (84) to compute the log ratio of these sets of taxa (Fig. 3d). Comparing the ratios of taxa in this way mitigates bias from the unknown total microbial load in each sample, and taking the log of this ratio gives equal weight to relative increases and decreases of taxa (83). Evaluation of the Songbird model against a baseline model obtained a pseudo-Q2 value of >0, suggesting that the model was not overfit. We found that coinfected individuals who had lifetime MDD had a significantly higher log ratio of set 1 to set 2 sOTUs than those who never had MDD (*t* test, *P* = 9.055e−06, *t* = −5.210, df = 34.183, Cohen’s D = 1.434), suggesting that they were associated with set 1. Several microbes that were associated with coinfected individuals with a lifetime history of MDD (set 1 microbes in Table S8) have also been previously identified as enriched in MDD, including *Enterobacteriaceae* (39) and *Alistepes* species (here, Alistepes onderdonkii) (2, 39, 85, 86), *Bacteroides* (39, 85), and *Parabacteroides* (here, Parabacteroides distasonis) (39). Likewise, coinfected individuals without lifetime MDD were enriched in several microbes (set 2 in Table S8) that were previously identified as being decreased in uninfected individuals with MDD, including *Dialister* spp. (39, 87), *Lachnospiraceae* (85), and *Ruminococcus* spp. (39).

### Conclusions.

This is to our knowledge the first study of the association between infection with HIV and HCV, depression, and the gut microbiome and metabolome. We performed 16S rRNA sequencing and liquid chromatography-mass spectrometry using stool samples from nearly 400 individuals and evaluated the data with state-of-the-art tools. We observed that although the gut microbiome of HIV-positive and -negative individuals differed, HCV had a large effect on the microbiome which warranted consideration in our study. The infection groups differed from each other in terms of both alpha and beta diversity in the full cohort as well as the MSM subgroups. Furthermore, we found that depression was associated with differences in the gut microbiome and metabolome only in HIV-HCV coinfected individuals. Coinfected individuals with a lifetime history of MDD were enriched in primary and secondary bile acids, as well as particular depression-related taxa. Importantly, our results suggest that microbiome and metabolome investigations in HIV-infected cohorts should carefully consider possible effects of HCV coinfections, which are not uncommon among people living with HIV.

People living with HIV and/or HCV are often burdened by a number of pharmaceutical interventions for treatment and management of their disease. Understanding the connection between the gut microbiome/metabolome and depression in patients with these comorbidities paves the way for microbiome-based interventions to treat depressive disorders (e.g., administration of probiotics or prebiotics, fecal transplants, or dietary interventions). *Bacteroides*, which we found to be associated with coinfected individuals with a history of MDD, can be decreased in the human gut by reducing consumption of animal protein and/or increasing carbohydrate intake (88). We also found that *Lachnospiraceae* are associated with non-MDD coinfected individuals. Our previous work suggests that *Lachnospiraceae* are present in people who consume fermented foods (89), again suggesting that dietary changes may be able to prevent or treat microbiome-based depressive disorders in these populations. While this study provides the foundation for more directed research, it has some limitations—particularly the lack of an HCV-monoinfected group, the small number of women, and the reduced sample sizes after forming MSM subsets. In future studies, it would also be of great interest to consider current MDD and other neurobehavioral or neuropsychiatric metrics in coinfected and monoinfected cohorts.

## MATERIALS AND METHODS

### Participant recruitment, sample processing, and sample selection.

This was a cross-sectional prospective observational cohort study of persons with or without HIV infection recruited from community sources, who agreed to undergo comprehensive neuromedical and neurobehavioral evaluations for NIH-funded studies at the HIV Neurobehavioral Research Program (HNRP; https://hnrp.hivresearch.ucsd.edu/) including the HIV Neurobehavioral Research Center (HNRC) at the University of California San Diego (UCSD). Study details can be found in references 90 and 91. Those who also agreed to submit stool samples for microbiome studies were included in the current analyses. A subset of participants also had positive serology for hepatitis C virus. The UCSD’s Human Research Protections Program (irb.ucsd.edu) approved all study procedures, and all participants provided written informed consent.

Exclusions were diagnoses of active substance use disorders and presence of an active, major psychiatric condition with current psychotic features or neurological conditions such as schizophrenia or epilepsy. If multiple stool samples were collected from participants, only the first time point was analyzed by 16S rRNA sequencing. A single time point per subject was additionally analyzed by high-performance liquid chromatography coupled to tandem mass spectrometry (HPLC-MS/MS). HIV and HCV infections were confirmed by a point-of-care vertical flow test (MedMira, Halifax, Nova Scotia, Canada). Participants were designated as follows: (i) “HIV monoinfected” if they tested positive for HIV but not HCV, (ii) “coinfected” if they tested positive for both HIV and HCV, or (iii) “uninfected” if they tested positive for neither HIV or HCV. Group characteristics were compared using *t* tests for all normally distributed continuous variables, Wilcoxon tests for nadir and current CD4, and chi-square tests for all nominal variables.

### Neuromedical and laboratory assessment.

All participants underwent a comprehensive neuromedical assessment, including a medical history that collected antiretroviral therapy (ART) and other medications, data to determine Centers for Disease Control (CDC) HIV disease staging, and specimen collection (blood, stool). Routine clinical chemistry panels, complete blood counts, rapid plasma reagin, and CD4^+^ T cells (flow cytometry) were performed at a Clinical Laboratory Improvement Amendments (CLIA)-certified medical center laboratory. HIV RNA was measured in plasma using reverse transcriptase PCR (Amplicor; Roche Diagnostics, Indianapolis, IN) with a lower limit of quantitation of 40 copies/ml.

### Evaluation of depression.

DSM-IV diagnosis of lifetime major depressive disorder was evaluated using the computer-assisted Composite International Diagnostic Interview (CIDI) (92), a structured instrument widely used in psychiatric research. Current self-reported depressed mood was assessed using the Beck Depression Inventory-II (BDI-II) (93). The BDI-II consists of 21 items that assess the severity of depression symptoms over the 2 weeks prior to assessment. The BDI-II total score ranges from 0 to 63 with higher scores denoting more severe depression symptoms. For analyses, we used the published cutoff of at least mild severity to define current self-reported depression (93).

### 16S rRNA gene sequencing.

DNA extraction and 16S rRNA amplicon sequencing were done using Earth Microbiome Project (EMP) standard protocols (http://www.earthmicrobiome.org/protocols-and-standards/16s). DNA was extracted with the Qiagen MagAttract PowerSoil DNA kit as previously described (94). Amplicon PCR was performed on the V4 region of the 16S rRNA gene using the primer pair 515f to 806r with Golay error-correcting barcodes on the reverse primer. Amplicons were barcoded and pooled in equal concentrations for sequencing. The amplicon pool was purified with the MO BIO UltraClean PCR cleanup kit and sequenced on the Illumina MiSeq sequencing platform. Sequence data were demultiplexed and minimally quality filtered using the Qiita defaults.

### 16S marker gene data analysis.

QIIME 2 v2020.2 (95) was used to rarefy to 2,500 sequences/sample and to generate pairwise unweighted UniFrac distances (52, 54, 96). Between group differences based on these distances were tested using PERMANOVA (97) and permuted *t* tests in QIIME 2. Alpha diversity (Shannon diversity [67]) was compared with a Kruskal-Wallis test.

Songbird v1.0.1 (83) in QIIME 2 version 2020.2 was used to identify feature ranks (parameters, –p-epochs, 10000; –batch-size, 5; –learning-rate, 1e−4; –min-sample-count, 1000; –min-feature-count, 0; –num-random-test-examples, 10), and Qurro v0.4.0 (84) was used to compute the log ratios of these ranked features. *t* tests and Cohen’s D were calculated to assess the significance (alpha = 0.05) and effect size of the log ratios.

### LC-MS/MS data acquisition.

Metabolomics sample processing and data acquisition protocols followed the standard Center for Microbiome Innovation’s seed grant project protocol to allow for comparison of this data set to many reference data sets and standards. Human fecal samples were transferred to clean 2-ml sample tubes (Qiagen catalog no. 990381), and the weights were recorded. The samples were then extracted in a solution of 1:1 methanol to water spiked with an internal standard of 1 μM sulfamethazine, using a 1:10 sample weight (in milligrams) to solvent volume (microliter) ratio. Using a Tissuelyser II (Qiagen), the samples were homogenized for 5 min at 25 Hz. This was followed by a 15-min centrifugation at 14,000 rpm. From the supernatant, 400 μl was transferred to a prelabeled 96-Well DeepWell plate, and the plates were concentrated using a CentriVap Benchtop Vacuum Concentrator (Labconco) for approximately 4 h. The dry plates were placed into a −80°C freezer until time for analysis.

The plates were resuspended in 150 μl of a 1:1 methanol-to-water solution with a 1 μM sulfadimethoxine internal standard solution. For metabolomics analysis, an ultrahigh performance liquid chromatography system (Thermo Dionex Ultimate 3000 UHPLC) coupled to an ultrahigh resolution quadrupole time of flight (qToF) mass spectrometer (Bruker Daltonics MaXis HD). For chromatographic separation, a Phenomenex Kinetex column (C_18_; 1.7 μm, 2.1 mm × 50 mm) was used, as this column has demonstrated robust separation of a large variety of the compounds within the parameters used. The mobile phase consisted of solvent A (100% LC-MS grade water with 0.1% formic acid) and solvent B (100% acetonitrile with 0.1% formic acid). Each sample was injected at a volume of 5 μl into a flow rate of 0.5 ml for the entire analysis. The 12-min chromatographic gradient began at 5% solvent B for the first minute, an increase to 100% solvent B from min 1 to min 11, a hold at 100% B until min 11.5, and back down to 5% solvent B reached at min 11.5. All data were collected using electrospray ionization in positive mode. Positive mode was selected in order to allow for spectral matches to be found using the GNPS spectral libraries, a majority of which were collected in positive ionization mode. Data-dependent acquisition was set to a scan range of 100 to 2,000 *m/z*.

### LC-MS/MS data analysis.

The raw data in Bruker (.d) format were lock mass corrected using hexakis (1H, 1H, 2H-difluoroethoxy)phosphazene (Synquest Laboratories, Alachua, FL) and were exported as .mzXML files using the Bruker Data Analysis software. Both the raw.d and the.mzXML files were uploaded to the UC San Diego mass spectrometry data repository MassIVE (https://massive.ucsd.edu/ProteoSAFe/static/massive.jsp). Feature detection was completed using MZmine version 2.37 software (98). Parameters can be found in Table S9 in the supplemental material. The resulting feature tables were exported as both a quantification file (.csv) and a spectral information file (.mgf) for analysis using the Global Natural Products Social Molecular Networking (GNPS) platform (99). All annotations obtained by GNPS fall under the Metabolomics Standards Initiative (MSI) (100) level 1, 2, or 3. The bile acids analyzed in Fig. 3a to c and Table S7 are MSI level 1 and 3. The level 1 annotations match the retention time of the bile acid standard run on the same gradient. The level 3 annotations do not have a retention time match to a standard and indicate a spectral match to that family of compounds. The annotation name listed represents the closest spectral match available in the GNPS libraries.

10.1128/mSystems.00465-20.9 TABLE S9 MZmine version 2.37 parameters used for feature detection on the metabolomics data. Download Table S9, CSV file, 0.00 MB. Copyright © 2020 Taylor et al. 2020 Taylor et al. This content is distributed under the terms of the Creative Commons Attribution 4.0 International license.

The quantification table and spectral information were analyzed using the GNPS feature-based molecular networking workflow (101). Parameters can be viewed via the job results page. For this data set, there were 1,911 unique MS/MS spectra of which 313 have spectral matches again the GNPS reference libraries (https://gnps.ucsd.edu/ProteoSAFe/libraries.jsp) including matches to drug and drug metabolite standards, bile acids, food-related compounds, and dipeptide molecules. The results reflect MSI level 2 or 3 annotations (100). For the statistical analyses, the MZmine-produced feature abundance table containing peak areas was inputted into the web-based MetaboAnalyst software (102). The data were normalized following the metabolomics data analysis protocols outlined in the previous metabolomics project (89), a normalization by quantile normalization and an auto scale. The normalized data were used to calculate a squareform matrix based on the Bray-Curtis distance metric which was inputted into a.qza format for use in QIIME2. All PERMANOVAs were run using the QIIME2 beta group significance command (95). The QIIME2 sample classifier command (default parameters) was used to assess the classification of lifetime MDD status in each infection group from the metabolomics data. The resulting features of importance and their GNPS annotations per group can be found in Tables S4, S5, and S6. The Cytoscape v3.7.2 software was used for all molecular networking visualizations (103). Individual feature level comparisons were completed using a Dunn’s test.

### Data availability.

The data generated in this study are available publicly in Qiita under the study ID 11135 (https://qiita.ucsd.edu/study/description/11135), and sequence data associated with this study have been deposited at EBI/ENA under accession number ERP122366. The raw experimental data are available at MassIVE (https://massive.ucsd.edu/), data set MSV000083664.
