# Correction for Taylor et al., “Depression in Individuals Coinfected with HIV and HCV Is Associated with Systematic Differences in the Gut Microbiome and Metabolome”

**DOI:** 10.1128/msystems.01305-24

**Published:** 2025-02-03

**Authors:** Bryn C. Taylor, Kelly C. Weldon, Ronald J. Ellis, Donald Franklin, Tobin Groth, Emily C. Gentry, Anupriya Tripathi, Daniel McDonald, Gregory Humphrey, MacKenzie Bryant, Julia Toronczak, Tara Schwartz, Michelli F. Oliveira, Robert Heaton, Igor Grant, Sara Gianella, Scott Letendre, Austin Swafford, Pieter C. Dorrestein, Rob Knight

## AUTHOR CORRECTION

Volume 5, no. 5, e00465-20, 2020, https://doi.org/10.1128/msystems.00465-20. Page 13, Acknowledgments: The following should be added as the last paragraph: “Rob Knight is a scientific advisory board member and consultant for BiomeSense, Inc., has equity, and receives income. He is a scientific advisory board member and has equity in GenCirq. He is a consultant and scientific advisory board member for DayTwo and receives income. He has equity in and acts as a consultant for Cybele. He is a co-founder of Biota, Inc., and has equity. He is a cofounder of Micronoma, has equity, and is a scientific advisory board member. The terms of these arrangements have been reviewed and approved by the University of California, San Diego, in accordance with its conflict-of-interest policies. Daniel McDonald is a consultant for BiomeSense, Inc., has equity, and receives income. The terms of these arrangements have been reviewed and approved by the University of California, San Diego, in accordance with its conflict-of-interest policies."

This correction does not change the results or the conclusions of this work.

